# Trends in Chronic Ischemic Heart Disease‐Related Mortality in Older Adults With Atrial Fibrillation (1999–2023): A CDC WONDER Database Analysis

**DOI:** 10.1002/clc.70267

**Published:** 2026-02-03

**Authors:** Muhammad Shaheer Bin Faheem, Syed Tawassul Hassan, Tehreem Asghar, Nafila Zeeshan, Sumaya Samadi

**Affiliations:** ^1^ Department of Medicine and Surgery Karachi Institute of Medical Sciences, KIMS Karachi Pakistan; ^2^ Karachi Medical and Dental College, KMDC Karachi Pakistan; ^3^ Akhtar Saeed Medical and Dental College Lahore Pakistan; ^4^ Dow University of Health Sciences Karachi Pakistan; ^5^ Kabul University of Medical Sciences “Abu Ali Ibn Sina” Kabul Afghanistan

**Keywords:** atrial fibrillation, CDC WONDER, ischemic heart disease

## Abstract

**Introduction:**

Atrial fibrillation (AF) is the most common and persistent type of arrhythmia that frequently co‐exists with chronic ischemic heart disease (IHD), increasing the risk of adverse cardiovascular outcomes and mortality. We aim to analyze chronic IHD‐related mortality trends in patients with AF from 1999 to 2023 in the United States (U.S.).

**Methods:**

Centers for Disease Control and Prevention's Wide‐Ranging Online Data for Epidemiologic Research (CDC WONDER) database was used to conduct a retrospective analysis of death records of adults (aged 65 ≤) with chronic IHD as the underlying cause and co‐existent AF as a contributing cause of death. Age‐adjusted mortality rates (AAMR) per 100 000 population and annual percent changes (APC) in age‐adjusted mortality rates were determined and measured across different demographics and geographies in the U.S.

**Results:**

AF was recorded in almost 460,196 deaths caused by chronic IHD. The AAMR increased from 38.2 in 1999 to 52.2 in 2023, showing a prominent increase shift from 2010 to 2023 (APC: 2.72). Recorded AAMR in Males (54.8) was doubled that of females (33.2), while a top AAMR of 44.7 was seen in non‐Hispanic (NH) Whites was doubled that of other racial/ethnic groups. Geographically, AAMR was higher in non‐metropolitan areas (43.3) and the Northeast region (42.8).

**Conclusions:**

Proper resource distribution and more targeted interventions are needed to address the rising trends in chronic IHD mortality among AF patients across different geographic and demographic groups.

## Graphical abstract

This study highlights rising age‐adjusted mortality rates from chronic ischemic heart disease in older adults with atrial fibrillation (1999–2023), with significant increases after 2010. Key disparities were observed by sex, race/ethnicity, urbanicity, and geography, emphasizing a need for targeted public health interventions

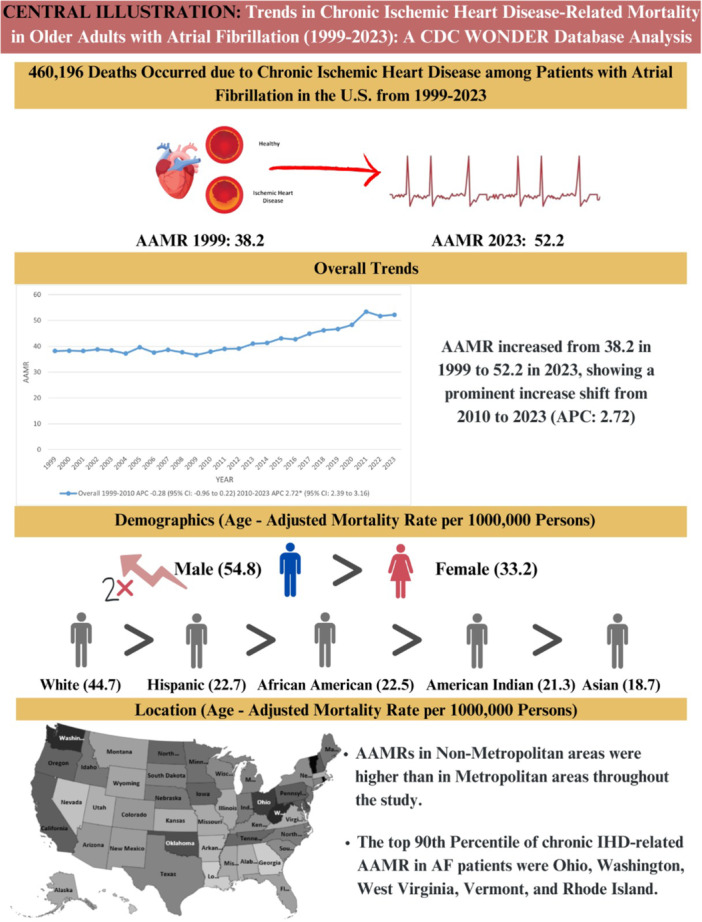

## 1 Introduction

AF is the most common sustained cardiac arrhythmia worldwide, and its prevalence continues to rise in aging populations [1]. The current national prevalence of diagnosed AF is estimated to be at least 10.55 million, markedly higher than earlier projections from studies conducted between 1996 and 1997, which estimated 3.3 million affected adults by 2020. This substantial increase underscores the urgent need for comprehensive prevention and management strategies [2]. Similarly, chronic IHD, classified under International Classification of Diseases, Tenth Revision (ICD‐10) code I25, remains a leading cause of death in the U.S., particularly among older adults [3].

AF and ischemic heart disease frequently coexist. According to studies, approximately 30% of patients with AF have coronary artery disease. Conversely, 15% of individuals with ischemic heart disease experience at least one episode of AF during their lifetime [4]. This bidirectional relationship can be attributed to various factors, including shared risk factors such as hypertension, diabetes, and obesity [5]. Chronic IHD can lead to atrial ischemia and fibrosis, which disrupt electrical conduction and promote the development of AF [6]. Additional contributing mechanisms include autonomic nervous system imbalance, increased systemic inflammation, and elevated oxidative stress [7]. Patients with coexisting AF and IHD have worse outcomes, including higher rates of stroke, heart failure, hospitalization, and mortality compared to those with either condition alone [8].

Our study aims to analyze national trends in mortality among U.S. adults aged 65 and older, where chronic IHD is listed as the underlying cause of death and AF as a contributing cause. Utilizing data from the CDC WONDER database, we examine temporal changes from 1999 to 2023 and assess demographic and geographic variations to better understand the evolving burden of these intersecting cardiovascular conditions.

## 2 Methods

### 2.1 Study Setting and Population

This study utilized the CDC WONDER database to examine trends in chronic IHD–related mortality among individuals with co‐existing AF in the United States from 1999 through 2023 [9]. Mortality data were obtained from the public‐use Multiple Cause of Death files, which are compiled from U.S. death certificates. Chronic IHD was identified using ICD‐10 code I25 as the underlying cause of death (UCOD), and AF was identified using ICD‐10 code I48 as a multiple (contributing) cause of death (MCOD). Adults aged 65 years and older were included in the analysis, given the higher burden of both conditions in this age group. Because the data are publicly available and de‐identified, this study was exempt from institutional review board (IRB) approval.

### 2.2 Data Extraction

Mortality data were extracted for all 50 U.S. states and the District of Columbia using the CDC WONDER interface. Demographic variables included age, sex, race, and ethnicity. Age was stratified into the following categories: 65–74, 75–84, and 85+ years. Racial and ethnic categories were consistent with CDC WONDER classifications and included: NH White, NH Black, Hispanic, NH Asian/Pacific Islander, and NH American Indian/Alaska Native. Geographic variables included U.S. Census Bureau regions (Northeast, Midwest, South, and West) and urban–rural classifications based on the National Center for Health Statistics (NCHS) Urban–Rural Classification Scheme [10]. All extracted data included both the number of deaths and corresponding population estimates for calculation of mortality rates.

### 2.3 Statistical Analysis

We calculated AAMRs per 100 000 population using the direct method and the U.S. 2000 Standard Population as the reference [11]. Crude mortality rates (CMRs) were also computed for context. Temporal trends were assessed using Joinpoint Regression Analysis Software (Version 5.0; National Cancer Institute), which identifies points where a statistically significant change in trend occurs. Annual percentage changes (APCs) in AAMRs and their corresponding 95% confidence intervals (CIs) were computed to evaluate changes in mortality rates over time [12]. Subgroup analyses were performed across sex, age groups, race/ethnicity, region, and urban–rural classifications to identify demographic and geographic disparities. A two‐tailed *P*‐value of < 0.05 was considered statistically significant. Data were analyzed and presented following CDC data usage guidelines to ensure appropriate interpretation of mortality trends.

## 3 Results

### 3.1 Proportional Mortality Rate Across Different Variables From 1999 to 2023

**FIGURE 1:**
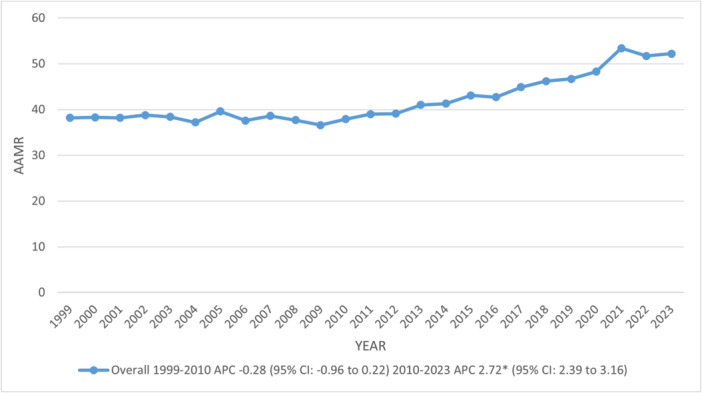
Overall, trends in chronic ischemic heart disease‐related age‐adjusted mortality rates per 100 000 among older adults with atrial fibrillation in the United States, 1999–2023. APC = Annual Percentage Change, CI = Confidence Interval. *Indicates that the Annual Percentage Change (APC) is significantly different from zero at α = 0.05.

A total of 460 196 deaths occurred due to CIHD among patients with AF. Females (49.34%) accounted higher proportion of deaths than males (50.66%), while among races/ethnicities, NH Whites (93.53%) were the highest, followed by NH African Americans (4.5%), Hispanic or Latino (3.57%) and NH Asian or Pacific Islander (1.61%) and lastly NH American Indians or Alaskan Natives (0.3%) contributed to least number of deaths. The majority of deaths were reported from nursing homes (30.99%), decedent's homes (29.41%), and Inpatient care (24.06%), with fewer in outpatient settings (6.23%), hospice facilities (4.31%), and other places (4.39%). Deaths were more dominant in metropolitan areas (80.98%) than in non‐metropolitan areas (19.02%), while regionally south (33.02%) reported higher deaths compared to other (Midwest: 23.39%, West: 22.89 and Northeast: 20.7%) regions (Supporting Information S1: Figure 1) (Supporting Information S1: Table 1).

### 3.2 Overall and Sex Stratified Age‐Adjusted Trends for Chronic Ischemic Heart Disease‐Related Mortality in Older Adults With Atrial Fibrillation, 1999–2023

**FIGURE 2:**
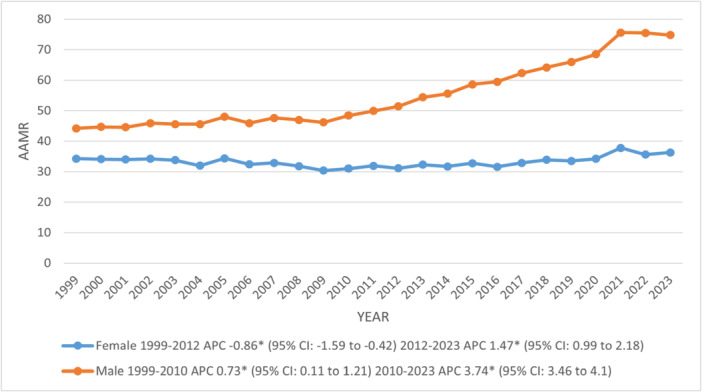
Trends in age‐adjusted mortality rates per 100 000 due to chronic ischemic heart disease, stratified by sex among older adults with atrial fibrillation in the United States, 1999–2023. APC = Annual Percentage Change, CI = Confidence Interval. *Indicates that the Annual Percentage Change (APC) is significantly different from zero at α = 0.05.

The AAMR increased from 38.2 in 1999 to 52.2 in 2023, with a marked rise observed between 2010 and 2023 (APC: 2.72; 95% CI: 2.39–3.16). Males (54.8) reported almost double the total AAMR of females at 33.2. However, the AAMR inclined significantly among both genders from 2010 in males and 2012 in females till 2023 at annual rates of 3.74% and 1.47%, respectively. Further, a notable decline was observed in females from 1999 to 2012 (95% CI: ‐0.86; 95% CI: ‐1.59 to ‐0.42) (Figures 1, 2) (Supporting Information S1: Tables 2, 3).

### 3.3 Race/Ethnicity Stratified

**FIGURE 3:**
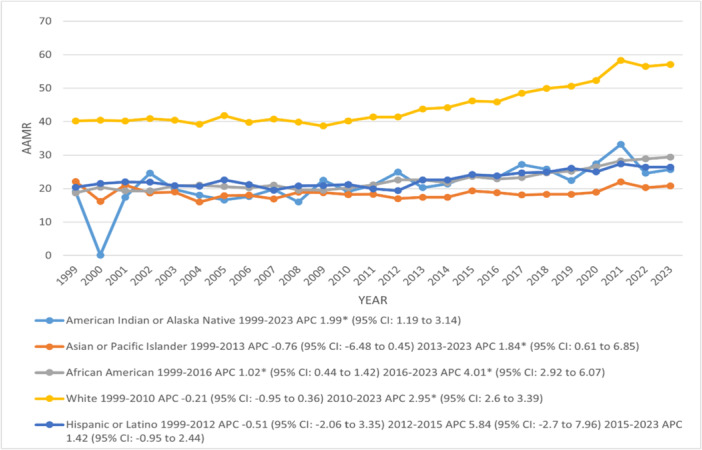
Trends in age‐adjusted mortality rates per 100 000 due to chronic ischemic heart disease, stratified by race/ethnicity among older adults with atrial fibrillation in the United States, 1999–2023. APC = Annual Percentage Change, CI = Confidence Interval. *Indicates that the Annual Percentage Change (APC) is significantly different from zero at α = 0.05.

The average mortality rate in NH whites (44.7) was more than doubled that of other races/ethnicities (Hispanic or Latino: 22.7, NH African American: 22.5, NH American Indian or Alaska Native: 21.3 and NH Asian or Pacific Islander: 18.7). The AAMRs increased significantly from 1999 to 2023 in NH American Indians or Alaska Natives (APC: 1.99; 95% CI: 1.19–3.14) and NH African Americans with a prominent increase showed by NH African Americans after 2016 (APC: 4.01; 95% CI: 2.92–6.07). NH Whites and NH Asians or Pacific Islanders experienced a sharp increase in AAMR from 2010, 2013 to 2023, with corresponding APCs of 2.95 and 1.84, respectively. No significant changes were seen in the AAMR of Hispanics or Latinos across the study period (Figure 3) (Supporting Information S1: Tables 3, 4).

### 3.4 Geographic Trends

**FIGURE 4:**
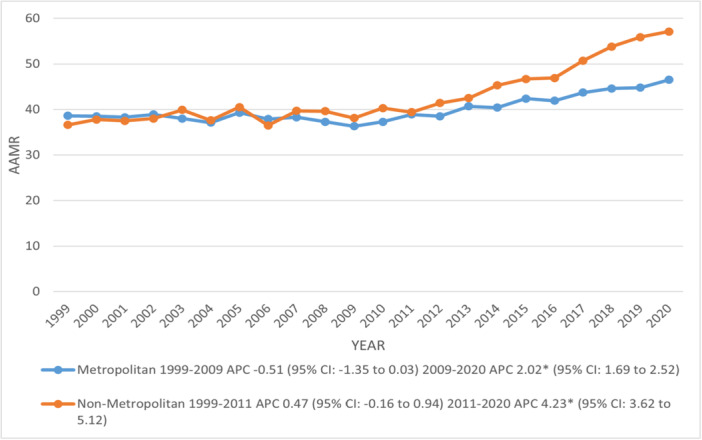
Trends in age‐adjusted mortality rates per 100 000 due to chronic ischemic heart disease, stratified by urbanization among older adults with atrial fibrillation in the United States, 1999–2020. APC = Annual Percentage Change, CI = Confidence Interval. *Indicates that the Annual Percentage Change (APC) is significantly different from zero at α = 0.05.

There were notable differences in AAMR among geographical subgroups, among which the AAMR of top 90th percentile States (Ohio: 56, Washington: 59.8, West Virginia: 60.3, Vermont: 67.6, and Rhode Island: 68.9) was doubled that of states (Georgia: 24.3, Louisiana: 24.3, Nevada: 26.7, Hawaii: 26.9 and Arkansas: 29.1) under lower 10th percentile. West (45.4) had the highest AAMR compared to the Midwest (42.8), Northeast (42.8), and South (38.5) regions. Comparatively, non‐metropolitan areas (43.3) documented higher AAMR than metropolitan areas (40.3), with AAMRs rising sharply in both metropolitan and non‐metropolitan areas after 2009 and 2011 till 2020, with corresponding APCs of 2.02 and 4.23, respectively (Figure 4) (Supporting Information S1: Figure 2) (Supporting Information S1: Tables 3–7).

## 4 Discussion

Using de‐identified national death certificate data from the CDC WONDER database, we identified several noteworthy trends. First, there has been a general increase in chronic IHD with AF mortality among adults in the United States over the past two decades. While this decline was evident in both sexes, males consistently exhibited higher mortality rates than females. Second, NH White older adults had the highest age‐ AAMR related to IHD and AFib, followed by NH Black individuals. Third, marked geographic disparities were evident, with rural regions experiencing the highest mortality rates. These findings have important implications for guiding targeted and equitable public health policy interventions.

The AAMR increased gradually from 1999 to 2023, with a marked rise observed in 2019, coinciding with the COVID‐19 pandemic. During this period, individuals with pre‐existing cardiovascular disease (CVD) risk factors experienced worse clinical outcomes and a greater demand for emergency cardiovascular care. Moreover, CDC data indicate that an estimated 20%–35% of COVID‐19‐related deaths occurred in patients with underlying CVD and associated risk factors [13]. Recent studies indicate that endothelial dysfunction, coagulopathy, and active inflammation were the most contributing factors to CVD in patients suffering from COVID‐19 [14]. Moreover, Wadhera et al. also reported a marked rise in mortality due to ischemic heart disease and associated disorders during the early phase of the COVID‐19 pandemic [15]. Individuals with preexisting health conditions, particularly those with IHD, were disproportionately affected, facing a significantly higher risk of severe illness and mortality. Beyond placing immense strain on healthcare systems, the pandemic exacerbated existing health disparities, potentially leading to delays or inadequacies in the management of chronic diseases [16].

AF, the most prevalent cardiac arrhythmia, and IHD frequently coexist due to their shared risk factors. These conditions exert a mutual influence, each contributing to the development and progression of the other through interconnected pathophysiological pathways, ultimately creating a complex and self‐perpetuating cycle, complicating the management [17]. As reported by Steensig et al., the mere presence of IHD rather than its severity as assessed by angiography was associated with a higher incidence of thromboembolic events, including ischemic stroke, transient ischemic attacks, systemic embolism, and death, in individuals with AF [17].

AF can trigger an inflammatory response that significantly accelerates atherosclerosis and contributes to IHD by enhancing left atrium activity. It also increases myocardial oxygen demand, which can outpace blood supply. AF and IHD are strongly associated with shared risk factors like hypertension, diabetes, obesity, and dyslipidemia [18, 19, 20].

Another potential reasoning suggests that chronic ischemia can induce structural and functional alterations in gap junction proteins, notably connexin 40 and connexin 43. These changes impair the uniform conduction of action potentials, leading to slowed and heterogeneous electrical propagation. Such conditions create a favorable substrate for AF through reentry mechanisms. Additionally, ischemia‐related necrosis of cardiomyocytes and their subsequent replacement by fibrotic tissue further support arrhythmogenic remodeling. The proinflammatory state linked to coronary ischemia, marked by elevated cytokines like interleukin‐6 (IL‐6), also contributes to atrial fibrosis by influencing the expression of matrix metalloproteinase‐2 (MMP‐2) [21, 22, 23].

Our analysis identified that AAMR associated with IHD and AF was two times greater in men compared to women. This finding contrasts with previous literature, which suggests that women have higher mortalities due to AF and IHD. Recent studies have indicated that women experience higher rates of complications and mortality associated with AF compared to men. Moreover, female patients are less likely to receive rhythm control interventions, including antiarrhythmic medications, cardioversion, and catheter ablation, resulting in higher deaths [24, 25, 26]. In contrast, findings from the Atherosclerosis Risk in Communities (ARIC) study, which tracked more than 15 000 individuals over nearly three decades, revealed a lifetime risk of AF of 36% in men and 30% in women, which aligns with our results [27]. Research has identified sex‐based differences in atrial electrophysiology. For example, the shortening of the atrial effective refractory period (ERP) in response to rapid atrial pacing is significantly less pronounced in premenopausal women compared to postmenopausal women and age‐matched men. This finding suggests that estrogen may provide a protective effect against AF in women [28, 29]. Body mass index (BMI) and obesity are well‐established risk factors for both AF and IHD. Previous research has identified sex‐related differences in the association between obesity and the long‐term risk of developing AF. While the Framingham Heart Study did not find statistically significant sex interactions, the effect estimates indicated a stronger association between obesity and AF incidence in men with IHD compared to women [30, 31].

Our data reveal significant racial and ethnic disparities among patients. NH White individuals exhibited the highest AAMR throughout the study period. This finding aligns with the established understanding that AF is more prevalent among White individuals [31]. In contrast, literature indicates that NH Black individuals have nearly double the mortality rate from IHD and AF compared to NH Whites, with lower medication adherence being linked to worse outcomes. Our analysis showed an increase in AAMRs for both White and Black populations, with the White population experiencing the most pronounced changes in mortality rates [32, 33]. Moreover, the opioid epidemic and rising rates of metabolic syndrome, particularly among middle‐aged and rural White populations, may be the culprit to offset improvements in cardiovascular mortality in these populations [34, 35]. Despite decades of progress toward equitable healthcare access for all racial groups in the United States, Black adults continue to experience inadequate management of chronic diseases, with CVD being the most significant concern; thus, new policies need to be made and implemented.

We observed significant regional variations in AAMRs, with the highest rates reported in the West, followed by the Midwest, Northeast, and South regions. Notably, West Virginia, Vermont, and Rhode Island exhibited particularly high AAMRs. Also, mortality rates were substantially higher in rural areas compared to urban regions. Rural regions often face delays in diagnosis and treatment due to limited healthcare resources, inadequate infrastructure, and transportation barriers. Additionally, factors such as a larger aging population, lower socioeconomic status, and reduced literacy levels are associated with higher mortality rates [36]. These healthcare disparities are also evident at the state level, where differences in the prevalence of risk factors, as well as prevention and early detection efforts, can be observed [36, 37]. Our findings highlight the urgent need for comprehensive, population‐based research in these areas to better understand the underlying causes of these disparities.

AF with IHD is currently one of the leading causes of death worldwide and is projected to maintain this position through 2050. Its global prevalence is expected to rise by 31.1% by the year 2060. Even when symptoms remain stable, IHD keeps evolving with marked accumulation of atherosclerotic plaques and changes in coronary circulation function [38]. Because this process can be influenced majorly by lifestyle factors such as diet, smoking, and physical inactivity, as well as by medications and revascularization procedures, there is a pressing necessity for enhanced research endeavors concentrated on the early detection and treatment of IHD. Moreover, culturally tailored interventions and extensive population‐based studies are critical for identifying the fundamental factors contributing to ongoing disparities in health outcomes. Initiatives such as the Million Hearts program could be expanded to include rural and nonmetropolitan populations, with an emphasis on risk factor mitigation, particularly in the management of hypertension and cholesterol levels. The National Rural Health Initiative plays a pivotal role in enhancing access to advanced cardiovascular care, which may include the implementation of telemedicine services and mobile clinics aimed at the early detection and management of IHD in underserved communities. Furthermore, frameworks such as Healthy People 2030 can inform state‐level strategies designed to diminish health disparities, focusing on equitable access to care for racial and ethnic minorities, who continue to endure elevated mortality rates. Enhancing access to primary percutaneous coronary intervention (PCI) facilities and providing incentives for the placement of cardiovascular specialists in underserved regions through federal programs, such as the National Health Service Corps, can significantly address geographic disparities. By synchronizing these strategies with existing initiatives, there exists substantial potential to alleviate inequities and enhance cardiovascular health outcomes across the United States.

## 5 Study Limitations

Our study had several limitations. Firstly, the data were based solely on ICD‐10 codes assigned by the WHO, which may be susceptible to omissions or inaccuracies. Additionally, we were unable to assess certain variables, such as the socioeconomic status of patients, due to a lack of available data, even though this is a critical factor in evaluating healthcare outcomes. The database also did not include laboratory or clinical findings, nor did it provide treatment histories for patients, which would have allowed for a more comprehensive analysis. Lastly, data on urbanization were not available after 2020, and state‐level data were collected only up to 2019 to avoid confounding effects from COVID‐19 deaths.

## 6 Conclusions

In conclusion, there has been a notable increase in mortality related to chronic IHD with AF among older adults in the United States over the past 2 decades. Although the overall trend shows improvement in some areas, males have consistently exhibited higher mortality rates compared to females. Second, NH White older adults demonstrated the highest AAMR for IHD and AF, followed by NH Black individuals. Third, significant geographic disparities persist, with rural areas bearing the highest mortality burden. These findings underscore the urgent need for targeted, equitable public health interventions to reduce cardiovascular mortality and address systemic health disparities.

## Author Contributions

Author Muhammad Shaheer Bin Faheem contributed to conceptualization, writing – original draft, writing – review and editing, data curation, visualization, formal analysis, and project administration. Author Syed Tawassul Hassan contributed to investigation, software, data curation, resources, formal analysis, and visualization. Author Tehreem Asghar contributed to validation, writing – original draft, and resources. Authors Nafila Zeeshan and Sumaya Samadi contributed to methodology and resources.

## Funding

The authors received no specific funding for this work.

## Ethics Statement

The authors have nothing to report.

## Consent

The authors have nothing to report.

## Conflicts of Interest

The authors declare no conflicts of interest.

## Supporting information


**Supplementary Figure 1.** Percent of total chronic ischemic heart disease‐related deaths by place of death among older adults with atrial fibrillation in the United States, 1999 to 2023. **Supplementary Figure 2.** Chronic ischemic heart disease‐related age‐adjusted mortality rates per 100,000, stratified by state among older adults with atrial fibrillation in the United States, 1999 to 2020. **Supplemental Table 1.** Absolute number and percentage of chronic ischemic heart disease‐related deaths among older adults with atrial fibrillation, stratified by the overall population, sex, race/ethnicity, place of death, urbanization, and U.S. region, 1999–2023. **Supplemental Table 2.** Overall and sex‐stratified chronic ischemic heart disease‐related age‐adjusted mortality rates per 100,000 among older adults with atrial fibrillation in the United States, 1999 to 2023. **Supplemental Table 3.** Annual percent change (APC) of chronic ischemic heart disease‐related age‐adjusted mortality rates per 100,000 among older adults with atrial fibrillation in the United States, 1999 to 2023. **Supplemental Table 4.** Race/ethnicity‐stratified chronic ischemic heart disease‐related age‐adjusted mortality rates per 100,000 among older adults with atrial fibrillation in the United States, 1999 to 2023. **Supplemental Table 5.** Urbanization‐stratified chronic ischemic heart disease‐related age‐adjusted mortality rates per 100,000 among older adults with atrial fibrillation in the United States, 1999 to 2020. **Supplemental Table 6.** Region‐stratified chronic ischemic heart disease‐related age‐adjusted mortality rates per 100,000 among older adults with atrial fibrillation in the United States, 1999 to 2023. **Supplemental Table 7.** State‐stratified chronic ischemic heart disease‐related age‐adjusted mortality rates per 100,000 and their respective percentiles among older adults with atrial fibrillation in the United States, 1999 to 2020.

## Data Availability

The datasets generated and/or analyzed during the current study are available within the manuscript and supplementary file.

## References

[1] S. S. Chugh , R. Havmoeller , K. Narayanan , et al., “Worldwide Epidemiology of Atrial Fibrillation: A Global Burden of Disease 2010 Study,” Circulation 129, no. 8 (2014): 837–847. 24345399 10.1161/CIRCULATIONAHA.113.005119 PMC4151302

[2] J. J. Noubiap , J. J. Tang , J. T. Teraoka , et al., “Minimum National Prevalence of Diagnosed Atrial Fibrillation Inferred From California Acute Care Facilities,” Journal of the American College of Cardiology 84, no. 16 (2024): 1501–1508. 39269390 10.1016/j.jacc.2024.07.014

[3] Centers for Disease Control and Prevention . Heart Disease Facts [Internet]. Atlanta (GA): CDC; 2024 [2025 Apr 30]. Available from: https://www.cdc.gov/heart-disease/data-research/facts-stats/index.html.

[4] F. W. A. Verheugt , J. M. Ten Berg , R. F. Storey , T. Cuisset , and C. B. Granger , “Antithrombotics,” Journal of the American College of Cardiology 74, no. 6 (2019): 699–711. 31277840 10.1016/j.jacc.2019.02.080

[5] M. Tomasdottir , C. Held , N. Hadziosmanovic , et al., “Risk Markers of Incident Atrial Fibrillation in Patients With Coronary Heart Disease,” American Heart Journal 233 (2021): 92–101. 33400910 10.1016/j.ahj.2020.12.016

[6] Amy. Does Ischemic Heart Disease Cause Atrial Fibrillation? Cardiovasc Dis Hub [Internet]. 2024 Oct 10 [cited 2025 May 1]. Available from: https://www.cardiovasculardiseasehub.com/archives/9675.

[7] Y. Xu , D. Sharma , G. Li , and Y. Liu , “Atrial Remodeling: New Pathophysiological Mechanism of Atrial Fibrillation,” Medical Hypotheses 80, no. 1 (2013): 53–56. 23148964 10.1016/j.mehy.2012.10.009

[8] E. S. Ford , U. A. Ajani , J. B. Croft , et al., “Explaining the Decrease in U.S. Deaths From Coronary Disease, 1980–2000,” New England Journal of Medicine 356, no. 23 (2007): 2388–2398. 17554120 10.1056/NEJMsa053935

[9] National Center for Health Statistics . Multiple Cause of Death 1999–2020 on CDC WONDER online database [Internet]. Hyattsville (MD): CDC; 2021 [2023 Sep 18]. Available from: http://wonder.cdc.gov/mcd-icd10.html.

[10] D. D. Ingram and S. J. Franco , *2013 NCHS Urban‐Rural Classification Scheme for Counties* (National Center for Health Statistics, 2014). 24776070

[11] R. N. Anderson and H. M. Rosenberg , “Age Standardization of Death Rates: Implementation of the Year 2000 Standard,” National Vital Statistics Reports 47, no. 3 (1998): 1–17. 9796247

[12] National Cancer Institute . Joinpoint Trend Analysis Software. Joinpoint Regression Program, version 2016 [Internet]. Bethesda (MD): Surveillance Research Program; 2016 [2023 Oct 14]. https://surveillance.cancer.gov/joinpoint/.

[13] R. Vasudeva , A. Challa , M. Al Rifai , et al., “Prevalence of Cardiovascular Diseases in COVID‐19‐Related Mortality in the United States,” Progress in Cardiovascular Diseases 74 (2022): 122–126. 36279944 10.1016/j.pcad.2022.09.002 PMC9585886

[14] S. X. Gu , T. Tyagi , K. Jain , et al., “Thrombocytopathy and Endotheliopathy: Crucial Contributors to COVID‐19 Thromboinflammation,” Nature Reviews Cardiology 18 (2021): 194–209, 10.1038/s41569-020-00469-1. 33214651 PMC7675396

[15] R. K. Wadhera , C. Shen , S. Gondi , S. Chen , D. S. Kazi , and R. W. Yeh , “Cardiovascular Deaths During the COVID‐19 Pandemic in the United States,” Journal of the American College of Cardiology 77 (2021): 159–169. 33446309 10.1016/j.jacc.2020.10.055 PMC7800141

[16] A. Ala , J. Wilder , N. L. Jonassaint , et al., “COVID‐19 and the Uncovering of Health Care Disparities in the United States, United Kingdom and Canada: Call to Action,” Hepatology Communications 5, no. 10 (2021): 1791–1800, 10.1002/hep4.1790. 34558861 PMC8426700

[17] K. Steensig , K. Olesen , T. Thim , et al., “Should the Presence or Extent of Coronary Artery Disease be Quantified in the CHA2DS2‐VASc Score in Atrial Fibrillation? A Report From the Western Denmark Heart Registry,” Thrombosis and Haemostasis 118 (2018): 2162–2170, 10.1055/s-0038-1675401. 30419601

[18] R. M. F. L. Da Silva , “Influence of Inflammation and Atherosclerosis in Atrial Fibrillation,” Current Atherosclerosis Reports 19 (2017): 2. 28102478 10.1007/s11883-017-0639-0

[19] F. Liang and Y. Wang , “Coronary Heart Disease and Atrial Fibrillation: A Vicious Cycle,” American Journal of Physiology‐Heart and Circulatory Physiology 320 (2021): H1–H12. 33185113 10.1152/ajpheart.00702.2020

[20] A. Krishnan , H. Sharma , D. Yuan , A. F. Trollope , and L. Chilton , “The Role of Epicardial Adipose Tissue in the Development of Atrial Fibrillation, Coronary Artery Disease and Chronic Heart Failure in the Context of Obesity and Type 2 Diabetes Mellitus: A Narrative Review,” Journal of Cardiovascular Development and Disease 9 (2022): 217. 35877579 10.3390/jcdd9070217 PMC9318726

[21] L. R. Luckett and R. M. Gallucci , “Interleukin‐6 (IL‐6) Modulates Migration and Matrix Metalloproteinase Function in Dermal Fibroblasts From IL‐6KO Mice,” British Journal of Dermatology 156 (2007): 1163–1171, 10.1111/j.1365-2133.2007.07867.x. 17441960

[22] C. Shu , W. Huang , Z. Zeng , et al., “Connexin 43 Is Involved in the Sympathetic Atrial Fibrillation in Canine and Canine Atrial Myocytes,” Anatolian Journal of Cardiology 18 (2017): 3–9, 10.14744/AnatolJCardiol.2017.7602. 28554986 PMC5512195

[23] E. Dupont , Y.‐S. Ko , S. Rothery , et al., “The Gap‐Junctional Protein Connexin40 Is Elevated in Patients Susceptible to Postoperative Atrial Fibrillation,” Circulation 103 (2001): 842–849, 10.1161/01.CIR.103.6.842. 11171793

[24] S. Stewart , C. L. Hart , D. J. Hole , and J. J. V. McMurray , “A Population‐Based Study of the Long‐Term Risks Associated With Atrial Fibrillation: 20‐Year Follow‐Up of the Renfrew/Paisley Study,” American Journal of Medicine 113 (2002): 359–364. 12401529 10.1016/s0002-9343(02)01236-6

[25] S. Westerman and N. Wenger , “Gender Differences in Atrial Fibrillation: A Review of Epidemiology, Management, and Outcomes,” Current Cardiology Reviews 15 (2019): 136–144. 30516110 10.2174/1573403X15666181205110624 PMC6520576

[26] X. T. Tian , Y. J. Xu , and Y. Q. Yang , “Gender Differences in Arrhythmias: Focused on Atrial Fibrillation,” Journal of Cardiovascular Translational Research 13 (2020): 85–96. 31637585 10.1007/s12265-019-09918-w

[27] L. Mou , F. L. Norby , L. Y. Chen , et al., “Lifetime Risk of Atrial Fibrillation by Race and Socioeconomic Status: ARIC Study (Atherosclerosis Risk in Communities),” Circulation: Arrhythmia and Electrophysiology 11, no. 7 (2018): e006350, 10.1161/CIRCEP.118.006350. 30002066 PMC6053683

[28] C. Magnussen , T. J. Niiranen , F. M. Ojeda , et al., “Sex Differences and Similarities in Atrial Fibrillation Epidemiology, Risk Factors, and Mortality in Community Cohorts: Results From the BiomarCaRE Consortium (Biomarker for Cardiovascular Risk Assessment in Europe),” Circulation 136 (2017): 1588–1597. 29038167 10.1161/CIRCULATIONAHA.117.028981 PMC5657474

[29] H. Bidoggia , J. P. Maciel , N. Capalozza , et al., “Sex Differences on the Electrocardiographic Pattern of Cardiac Repolarization: Possible Role of Testosterone,” American Heart Journal 140 (2000): 678–683. 11011345 10.1067/mhj.2000.109918

[30] T. J. Wang , “Obesity and the Risk of New‐Onset Atrial Fibrillation,” Journal of the American Medical Association 292 (2004): 2471–2477, 10.1001/jama.292.20.2471. 15562125

[31] W. H. Sauer and P. C. Zei , “Atrial Fibrillation,” in Harrison's Principles of Internal Medicine 21ed, eds. J. Loscalzo , A. Fauci , D. Kasper , S. Hauser , D. Longo , and J. L. Jameson , (McGraw‐Hill Education, 2022).

[32] D. Islek , A. Alonso , W. Rosamond , et al., Racial Differences in Fatal Out‐of‐Hospital Coronary Heart Disease and the Role of Income in the Atherosclerosis Risk in Community's Cohort Study (1987 to 2017). 10.1016/j.amjcard.2023.01.042 PMC10079596 36914508

[33] L. D. Colantonio , C. M. Gamboa , J. S. Richman , et al., “Black‐White Differences in Incident Fatal, Nonfatal, and Total Coronary Heart Disease,” Circulation 136, no. 2 (2017): 152–166. 28696265 10.1161/CIRCULATIONAHA.116.025848 PMC5551431

[34] M. J. Alexander , M. V. Kiang , and M. Barbieri , “Trends in Black and White Opioid Mortality in the United States, 1979–2015,” Epidemiology 29, no. 5 (2018): 707–715, 10.1097/EDE.0000000000000858. 29847496 PMC6072374

[35] Prevalence of the Metabolic Syndrome Among US Adults: Findings From the Third National Health and Nutrition Examination Survey, Cardiology | JAMA | JAMA Network, accessed February 10, 2025, https://jamanetwork.com/journals/jama/fullarticle/194559. 10.1001/jama.287.3.356 11790215

[36] J. P. Leider , M. Meit , J. M. McCullough , et al., “The State of Rural Public Health: Enduring Needs in a New Decade,” American Journal of Public Health 110, no. 9 (2020): 1283–1290. 32673103 10.2105/AJPH.2020.305728 PMC7427223

[37] S. S. Coughlin , C. Clary , J. A. Johnson , et al., “Continuing Challenges in Rural Health in the United States,” Journal of Environment and Health Sciences 5, no. 2 (2019): 90–92. 32104722 PMC7043306

[38] R. Hajhosseiny , C. Munoz , G. Cruz , et al., “Coronary Magnetic Resonance Angiography in Chronic Coronary Syndromes,” Frontiers in Cardiovascular Medicine 8 (2021): 682964. 10.3389/fcvm.2021.682924 PMC8416045 34485397

