# Supplementary material for: Trends in Chronic Ischemic Heart Disease‐Related Mortality in Older Adults With Atrial Fibrillation (1999–2023): A CDC WONDER Database Analysis
Source: Clin Cardiol. 2026 Feb 3;49(2):e70267. doi: 10.1002/clc.70267 (PMC12865863; doi:10.1002/clc.70267)
Supplement: Supplementary file 1 — Supplementary Figure 1. Percent of total chronic ischemic heart disease‐related deaths by place of death among older adults with atrial fibrillation in the United States, 1999 to 2023. Supplementary Figure 2. Chronic ischemic heart disease‐related age‐adjusted mortality rates per 100,000, stratified by state among older adults with atrial fibrillation in the United States, 1999 to 2020. Supplemental Table 1. Absolute number and percentage of chronic ischemic heart disease‐related deaths among older adults with atrial fibrillation, stratified by the overall population, sex, race/ethnicity, place of death, urbanization, and U.S. region, 1999–2023. Supplemental Table 2. Overall and sex‐stratified chronic ischemic heart disease‐related age‐adjusted mortality rates per 100,000 among older adults with atrial fibrillation in the United States, 1999 to 2023. Supplemental Table 3. Annual percent change (APC) of chronic ischemic heart disease‐related age‐adjusted mortality rates per 100,000 among older adults with atrial fibrillation in the United States, 1999 to 2023. Supplemental Table 4. Race/ethnicity‐stratified chronic ischemic heart disease‐related age‐adjusted mortality rates per 100,000 among older adults with atrial fibrillation in the United States, 1999 to 2023. Supplemental Table 5. Urbanization‐stratified chronic ischemic heart disease‐related age‐adjusted mortality rates per 100,000 among older adults with atrial fibrillation in the United States, 1999 to 2020. Supplemental Table 6. Region‐stratified chronic ischemic heart disease‐related age‐adjusted mortality rates per 100,000 among older adults with atrial fibrillation in the United States, 1999 to 2023. Supplemental Table 7. State‐stratified chronic ischemic heart disease‐related age‐adjusted mortality rates per 100,000 and their respective percentiles among older adults with atrial fibrillation in the United States, 1999 to 2020. [file CLC-49-e70267-s001.docx]

| **Variables** | **Deaths** | **% of Total Deaths** |
| --- | --- | --- |
| Overall | 460196 | 100 |
| **Gender** | | |
| Female | 227047 | 49.34 |
| Male | 233149 | 50.66 |
| **Race/Ethnicity** | | |
| NH American Indian or Alaskan Native | 1399 | 0.3 |
| NH Asian or Pacific Islander | 7401 | 1.61 |
| NH African American | 20689 | 4.5 |
| NH White | 430408 | 93.53 |
| Hispanic or Latino | 16411 | 3.57 |
| **Place of Deaths** | | |
| Medical Facility - Inpatient | 170116 | 24.06 |
| Medical Facility - Outpatient or ER | 63847 | 6.23 |
| Medical Facility - Dead on Arrival | 4926 | 0.43 |
| Decedent's home | 83984 | 29.41 |
| Hospice facility | 5764 | 4.31 |
| Nursing home/long term care | 55876 | 30.99 |
| Other | 10719 | 4.39 |
| **Urbanization** | | |
| Metropolitan Areas | 306757 | 80.98 |
| Non-Metropolitan Areas | 72039 | 19.02 |
| **Regions** | | |
| Northeast | 95253 | 20.7 |
| Midwest | 107619 | 23.39 |
| South | 151974 | 33.02 |
| West | 105350 | 22.89 |
| **NH: Non-Hispanic** | | |

**Supplemental Table 1.** Absolute number and percentage of chronic ischemic heart disease-related deaths among older adults with atrial fibrillation, stratified by the overall population, sex, race/ethnicity, place of death, urbanization, and U.S. region, 1999–2023.


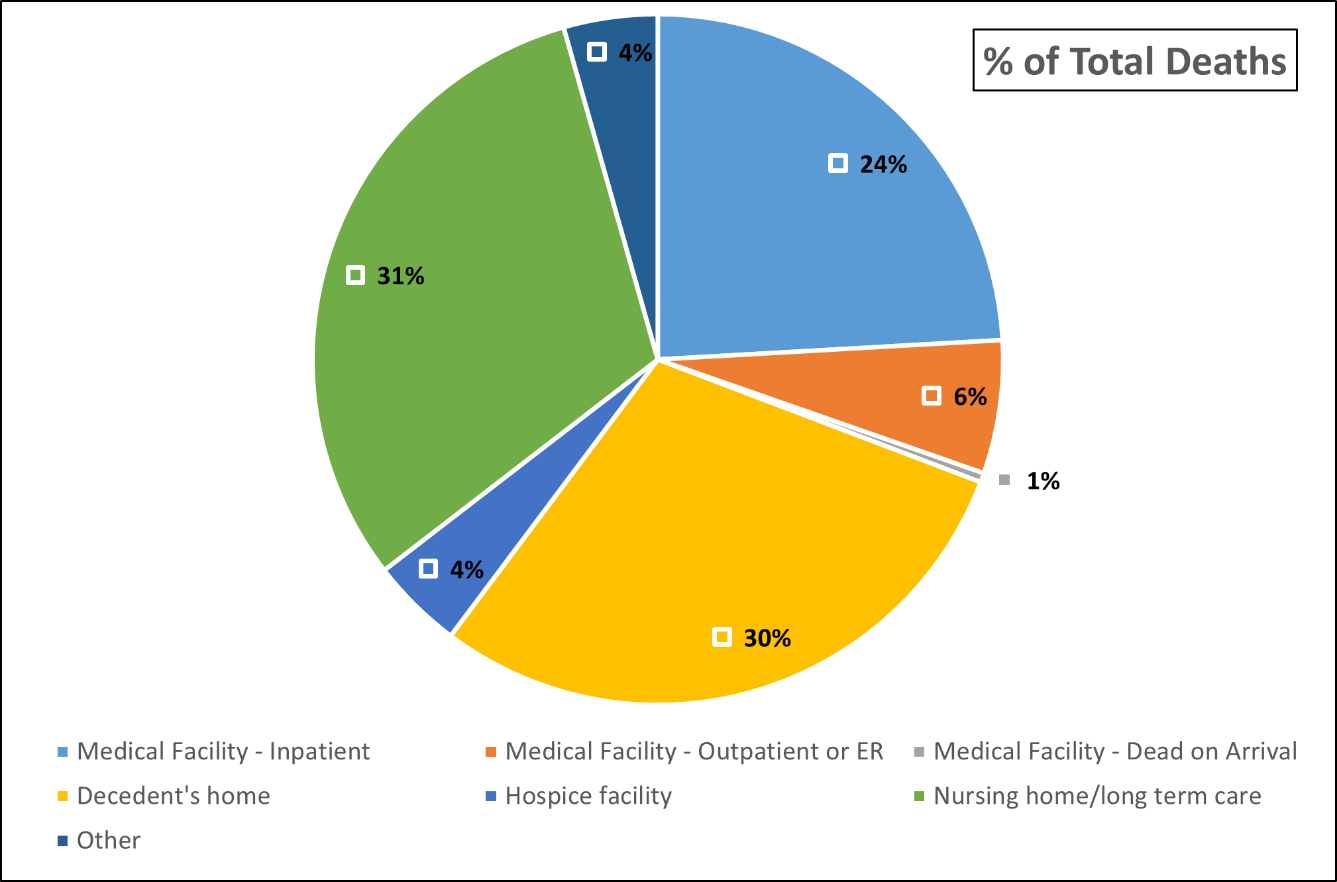


**Supplementary Figure 1.** Percent of total chronic ischemic heart disease-related deaths by place of death among older adults with atrial fibrillation in the United States, 1999 to 2023.

| **Age-Adjusted Rate /100,000 (95% CI)** | | | |
| --- | --- | --- | --- |
| **Year** | **Overall** | **Male** | **Female** |
| 1999 | 38.2 (37.5 - 38.9) | 44.2 (42.9 - 45.4) | 34.3 (33.5 - 35) |
| 2000 | 38.3 (37.7 - 39) | 44.7 (43.5 - 45.9) | 34.1 (33.3 - 34.9) |
| 2001 | 38.2 (37.5 - 38.8) | 44.6 (43.4 - 45.8) | 34 (33.2 - 34.7) |
| 2002 | 38.8 (38.2 - 39.5) | 45.9 (44.7 - 47.1) | 34.2 (33.5 - 35) |
| 2003 | 38.4 (37.8 - 39.1) | 45.6 (44.4 - 46.8) | 33.8 (33 - 34.5) |
| 2004 | 37.2 (36.6 - 37.8) | 45.6 (44.4 - 46.7) | 32 (31.3 - 32.7) |
| 2005 | 39.6 (38.9 - 40.2) | 48 (46.8 - 49.1) | 34.4 (33.6 - 35.1) |
| 2006 | 37.6 (37 - 38.2) | 45.9 (44.7 - 47) | 32.4 (31.7 - 33.1) |
| 2007 | 38.6 (38 - 39.2) | 47.6 (46.5 - 48.8) | 32.9 (32.2 - 33.6) |
| 2008 | 37.7 (37.1 - 38.3) | 47 (45.8 - 48.1) | 31.8 (31.1 - 32.5) |
| 2009 | 36.6 (36 - 37.2) | 46.2 (45.2 - 47.3) | 30.4 (29.8 - 31.1) |
| 2010 | 37.9 (37.3 - 38.5) | 48.4 (47.3 - 49.5) | 31 (30.3 - 31.7) |
| 2011 | 39 (38.4 - 39.6) | 49.9 (48.8 - 51) | 31.9 (31.3 - 32.6) |
| 2012 | 39.1 (38.5 - 39.7) | 51.4 (50.3 - 52.5) | 31.1 (30.4 - 31.7) |
| 2013 | 41 (40.4 - 41.6) | 54.4 (53.3 - 55.5) | 32.3 (31.6 - 32.9) |
| 2014 | 41.3 (40.7 - 41.9) | 55.6 (54.5 - 56.7) | 31.7 (31 - 32.3) |
| 2015 | 43.1 (42.5 - 43.7) | 58.6 (57.5 - 59.7) | 32.8 (32.1 - 33.5) |
| 2016 | 42.7 (42.2 - 43.3) | 59.5 (58.4 - 60.6) | 31.6 (30.9 - 32.2) |
| 2017 | 44.9 (44.3 - 45.5) | 62.3 (61.2 - 63.4) | 32.9 (32.2 - 33.6) |
| 2018 | 46.2 (45.6 - 46.8) | 64.2 (63 - 65.3) | 33.9 (33.2 - 34.5) |
| 2019 | 46.7 (46.1 - 47.3) | 66 (64.9 - 67.1) | 33.5 (32.8 - 34.1) |
| 2020 | 48.3 (47.7 - 48.9) | 68.5 (67.4 - 69.6) | 34.2 (33.5 - 34.8) |
| 2021 | 53.4 (52.7 - 54) | 75.6 (74.4 - 76.8) | 37.8 (37.1 - 38.5) |
| 2022 | 51.7 (51.1 - 52.3) | 75.5 (74.3 - 76.7) | 35.6 (35 - 36.3) |
| 2023 | 52.2 (51.6 - 52.8) | 74.8 (73.7 - 76) | 36.3 (35.6 - 37) |
| **Total** | 41.9 (41.3 - 42.5) | 54.8 (53.7 - 55.9) | 33.2 (32.5 - 33.9) |

**Supplemental Table 2.** Overall and sex-stratified chronic ischemic heart disease-related age-adjusted mortality rates per 100,000 among older adults with atrial fibrillation in the United States, 1999 to 2023.


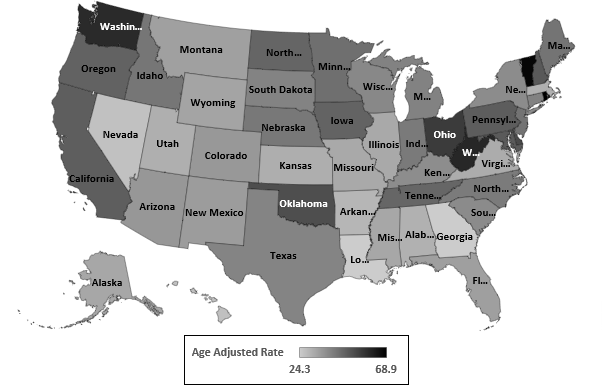


**Supplementary Figure 2.** Chronic ischemic heart disease-related age-adjusted mortality rates per 100,000, stratified by state among older adults with atrial fibrillation in the United States, 1999 to 2020.

| **Year Interval** | **APC (95% CI)** |
| --- | --- |
| **Overall** | |
| 1999-2010 | -0.28 (-0.96 - 0.22) |
| 2010-2023 | 2.72* (2.39 - 3.16) |
| **Gender** | |
| **Female** | |
| 1999-2012 | -0.86* (-1.59 - -0.42) |
| 2012-2023 | 1.47* (0.99 - 2.18) |
| **Male** | |
| 1999-2010 | 0.73* (0.11 - 1.21) |
| 2010-2023 | 3.74* (3.46 - 4.1) |
| **Race** | |
| **NH American Indian or Alaska Native** | |
| 1999-2023 | 1.99* (1.19 - 3.14) |
| **NH Asian or Pacific Islander** | |
| 1999-2013 | -0.76 (-6.48 - 0.45) |
| 2013-2023 | 1.84* (0.61 - 6.85) |
| **NH African American** | |
| 1999-2016 | 1.02* (0.44 - 1.42) |
| 2016-2023 | 4.01* (2.92 - 6.07) |
| **NH White** | |
| 1999-2010 | -0.21 (-0.95 - 0.36) |
| 2010-2023 | 2.95* (2.6 - 3.39) |
| **Hispanic or Latino** | |
| 1999-2012 | -0.51 (-2.06 - 3.35) |
| 2012-2015 | 5.84 (-2.7 - 7.96) |
| 2015-2023 | 1.42 (-0.95 - 2.44) |
| **Urbanization** | |
| **Metropolitan** | |
| 1999-2009 | -0.51 (-1.35 - 0.03) |
| 2009-2020 | 2.02* (1.69 - 2.52) |
| **Non-Metropolitan** | |
| 1999-2011 | 0.47 (-0.16 - 0.94) |
| 2011-2020 | 4.23* (3.62 - 5.12) |
| **NH: Non-Hispanic; APC: Annual Percent Change** | |

**Supplemental Table 3.** Annual percent change (APC) of chronic ischemic heart disease-related age-adjusted mortality rates per 100,000 among older adults with atrial fibrillation in the United States, 1999 to 2023.

| **Age-Adjusted Rate /100,000 (95% CI)** | | | | | |
| --- | --- | --- | --- | --- | --- |
| **Year** | **NH American Indian or Alaska Native** | **NH Asian or Pacific Islander** | **NH African American** | **NH White** | **Hispanics or Latino** |
| 1999 | 19.1 (11.8 - 29.2) | 22.1 (18.3 - 25.9) | 18.7 (17.1 - 20.4) | 40.2 (39.5 - 40.9) | 20.4 (18 - 22.9) |
| 2000 | 0.1 (5.9 - 18.2) | 16.2 (13.2 - 19.3) | 20.4 (18.7 - 22.1) | 40.4 (39.7 - 41.1) | 21.5 (19 - 24) |
| 2001 | 17.4 (11.1 - 25.9) | 21.1 (17.7 - 24.5) | 19.3 (17.6 - 20.9) | 40.2 (39.5 - 40.9) | 22 (19.6 - 24.4) |
| 2002 | 24.6 (16.9 - 34.5) | 18.7 (15.6 - 21.8) | 19.3 (17.7 - 21) | 40.9 (40.2 - 41.6) | 21.9 (19.5 - 24.3) |
| 2003 | 19.8 (13.3 - 28.5) | 19 (15.9 - 22) | 20.8 (19.1 - 22.5) | 40.4 (39.7 - 41.1) | 20.9 (18.7 - 23.2) |
| 2004 | 18 (11.9 - 26.2) | 16 (13.3 - 18.7) | 21 (19.3 - 22.7) | 39.2 (38.5 - 39.8) | 20.7 (18.5 - 22.9) |
| 2005 | 16.6 (10.7 - 24.8) | 17.9 (15.2 - 20.6) | 20.6 (18.9 - 22.3) | 41.8 (41.1 - 42.5) | 22.6 (20.4 - 24.8) |
| 2006 | 17.6 (11.8 - 25.3) | 18 (15.4 - 20.6) | 20.2 (18.5 - 21.8) | 39.8 (39.1 - 40.4) | 21.2 (19.1 - 23.2) |
| 2007 | 19.8 (13.4 - 28.1) | 16.9 (14.4 - 19.4) | 21 (19.4 - 22.7) | 40.8 (40.1 - 41.4) | 19.5 (17.5 - 21.4) |
| 2008 | 16 (10.6 - 23.3) | 18.9 (16.3 - 21.4) | 19.6 (18 - 21.1) | 39.9 (39.3 - 40.6) | 20.8 (18.8 - 22.7) |
| 2009 | 22.5 (16.2 - 30.4) | 18.8 (16.4 - 21.3) | 19.5 (18 - 21.1) | 38.7 (38 - 39.3) | 20.9 (19 - 22.7) |
| 2010 | 19.1 (13.5 - 26.3) | 18.2 (15.8 - 20.5) | 20.2 (18.6 - 21.7) | 40.2 (39.5 - 40.8) | 21.2 (19.3 - 23) |
| 2011 | 21 (15.3 - 28.2) | 18.3 (16.1 - 20.5) | 21.1 (19.5 - 22.7) | 41.4 (40.7 - 42) | 19.9 (18.2 - 21.6) |
| 2012 | 24.9 (18.8 - 32.2) | 17 (15 - 19.1) | 22.6 (21 - 24.2) | 41.4 (40.8 - 42.1) | 19.4 (17.8 - 21.1) |
| 2013 | 20.3 (15.1 - 26.8) | 17.4 (15.4 - 19.4) | 22.6 (21.1 - 24.2) | 43.8 (43.1 - 44.5) | 22.6 (20.9 - 24.3) |
| 2014 | 21.4 (16.1 - 27.8) | 17.4 (15.5 - 19.4) | 21.7 (20.2 - 23.2) | 44.2 (43.6 - 44.9) | 22.6 (20.9 - 24.2) |
| 2015 | 24.1 (18.6 - 30.7) | 19.3 (17.3 - 21.2) | 23.6 (22.1 - 25.1) | 46.2 (45.5 - 46.9) | 24.2 (22.6 - 25.9) |
| 2016 | 23.3 (18.1 - 29.5) | 18.8 (16.9 - 20.7) | 22.9 (21.4 - 24.3) | 45.9 (45.3 - 46.6) | 23.8 (22.2 - 25.4) |
| 2017 | 27.2 (21.7 - 33.6) | 18.1 (16.3 - 19.9) | 23.3 (21.9 - 24.8) | 48.5 (47.8 - 49.1) | 24.7 (23.1 - 26.3) |
| 2018 | 25.8 (20.7 - 31.7) | 18.3 (16.6 - 20.1) | 24.7 (23.2 - 26.2) | 49.9 (49.2 - 50.5) | 24.9 (23.3 - 26.5) |
| 2019 | 22.4 (17.7 - 28) | 18.3 (16.6 - 20) | 25.2 (23.7 - 26.6) | 50.6 (49.9 - 51.2) | 26.1 (24.5 - 27.6) |
| 2020 | 27.4 (22.1 - 32.7) | 18.9 (17.2 - 20.6) | 26.5 (25.1 - 28) | 52.3 (51.7 - 53) | 25 (23.5 - 26.5) |
| 2021 | 33.2 (27.2 - 39.3) | 22 (20.1 - 23.8) | 28.3 (26.8 - 29.9) | 58.3 (57.6 - 59) | 27.4 (25.9 - 29) |
| 2022 | 24.6 (19.9 - 30.2) | 20.3 (18.6 - 22) | 28.9 (27.3 - 30.4) | 56.5 (55.8 - 57.2) | 26.4 (24.9 - 27.9) |
| 2023 | 25.7 (20.8 - 30.6) | 20.8 (19.1 - 22.5) | 29.4 (27.9 - 30.9) | 57.1 (56.4 - 57.8) | 26.4 (24.9 - 27.9) |
| **Total** | 21.3 (16 - 28.9) | 18.7 (16.3 - 21) | 22.5 (20.9 - 24) | 44.7 (44.1 - 45.4) | 22.7 (20.8 - 24.5) |
| **NH: Non-Hispanic** | | | | | |

**Supplemental Table 4.** Race/ethnicity-stratified chronic ischemic heart disease-related age-adjusted mortality rates per 100,000 among older adults with atrial fibrillation in the United States, 1999 to 2023.

| **Age-Adjusted Rate /100,000 (95% CI)** | | |
| --- | --- | --- |
| **Year** | **Metropolitan** | **Non-Metropolitan** |
| 1999 | 38.6 (37.9 - 39.3) | 36.6 (35.2 - 38.1) |
| 2000 | 38.5 (37.8 - 39.2) | 37.8 (36.3 - 39.3) |
| 2001 | 38.3 (37.6 - 39.1) | 37.5 (36.1 - 39) |
| 2002 | 38.9 (38.2 - 39.7) | 38 (36.6 - 39.5) |
| 2003 | 38 (37.3 - 38.7) | 39.9 (38.5 - 41.4) |
| 2004 | 37.1 (36.4 - 37.8) | 37.6 (36.1 - 39) |
| 2005 | 39.3 (38.6 - 40.1) | 40.5 (39 - 42) |
| 2006 | 37.9 (37.2 - 38.6) | 36.5 (35.1 - 37.9) |
| 2007 | 38.3 (37.7 - 39) | 39.7 (38.3 - 41.2) |
| 2008 | 37.3 (36.6 - 38) | 39.6 (38.2 - 41) |
| 2009 | 36.3 (35.7 - 37) | 38.1 (36.7 - 39.5) |
| 2010 | 37.3 (36.6 - 37.9) | 40.3 (38.9 - 41.7) |
| 2011 | 38.9 (38.3 - 39.6) | 39.4 (37.9 - 40.8) |
| 2012 | 38.5 (37.9 - 39.2) | 41.4 (39.9 - 42.8) |
| 2013 | 40.7 (40.1 - 41.4) | 42.5 (41.1 - 43.9) |
| 2014 | 40.4 (39.8 - 41) | 45.3 (43.8 - 46.8) |
| 2015 | 42.4 (41.7 - 43) | 46.7 (45.2 - 48.2) |
| 2016 | 41.9 (41.3 - 42.5) | 46.9 (45.5 - 48.4) |
| 2017 | 43.7 (43.1 - 44.3) | 50.7 (49.1 - 52.2) |
| 2018 | 44.6 (43.9 - 45.2) | 53.8 (52.3 - 55.4) |
| 2019 | 44.8 (44.2 - 45.4) | 55.9 (54.3 - 57.5) |
| 2020 | 46.5 (45.9 - 47.2) | 57.1 (55.5 - 58.6) |
| **Total** | 40.3 (40.1 - 40.4) | 43.3 (43 - 43.6) |

**Supplemental Table 5.** Urbanization-stratified chronic ischemic heart disease-related age-adjusted mortality rates per 100,000 among older adults with atrial fibrillation in the United States, 1999 to 2020.

| **Age-Adjusted Rate /100,000 (95% CI)** | | | | |
| --- | --- | --- | --- | --- |
| **Year** | **Northeast** | **Midwest** | **South** | **West** |
| 1999 | 42.8 (41.3 - 44.3) | 36.4 (35.1 - 37.7) | 32.9 (31.9 - 34) | 44.7 (43.1 - 46.4) |
| 2000 | 42.4 (40.9 - 43.9) | 36.3 (35 - 37.6) | 33.6 (32.6 - 34.6) | 44.6 (43 - 46.2) |
| 2001 | 43.6 (42.1 - 45.1) | 35.8 (34.5 - 37.1) | 33.3 (32.3 - 34.4) | 43.6 (42 - 45.1) |
| 2002 | 43.3 (41.8 - 44.7) | 37.2 (35.9 - 38.5) | 34.2 (33.2 - 35.3) | 43.5 (42 - 45.1) |
| 2003 | 42.9 (41.4 - 44.3) | 36.5 (35.2 - 37.8) | 33.7 (32.7 - 34.7) | 43.9 (42.4 - 45.4) |
| 2004 | 42 (40.6 - 43.4) | 36.2 (35 - 37.5) | 32.5 (31.5 - 33.5) | 41.1 (39.6 - 42.5) |
| 2005 | 42 (40.6 - 43.4) | 39.3 (38 - 40.5) | 35.7 (34.7 - 36.7) | 43.7 (42.3 - 45.2) |
| 2006 | 39.6 (38.2 - 41) | 37.3 (36.1 - 38.6) | 33.2 (32.2 - 34.2) | 43.3 (41.9 - 44.8) |
| 2007 | 40.4 (39 - 41.8) | 38.7 (37.4 - 40) | 35 (34 - 36) | 42.5 (41 - 43.9) |
| 2008 | 39.9 (38.5 - 41.2) | 39.3 (38 - 40.6) | 32.8 (31.9 - 33.8) | 41.7 (40.3 - 43.1) |
| 2009 | 37.5 (36.2 - 38.8) | 37.3 (36.1 - 38.5) | 33.2 (32.3 - 34.2) | 40.3 (38.9 - 41.7) |
| 2010 | 40.3 (39 - 41.7) | 38 (36.8 - 39.2) | 33.6 (32.6 - 34.5) | 42.1 (40.7 - 43.4) |
| 2011 | 42.1 (40.8 - 43.5) | 39.2 (37.9 - 40.4) | 34.5 (33.6 - 35.4) | 43 (41.7 - 44.4) |
| 2012 | 41.3 (40 - 42.7) | 40.4 (39.1 - 41.6) | 35.3 (34.4 - 36.3) | 41.5 (40.2 - 42.8) |
| 2013 | 42.7 (41.4 - 44.1) | 41.6 (40.4 - 42.9) | 36.7 (35.7 - 37.6) | 45.7 (44.3 - 47) |
| 2014 | 42.6 (41.3 - 43.9) | 41.4 (40.2 - 42.7) | 38.2 (37.3 - 39.2) | 44.7 (43.4 - 46) |
| 2015 | 43.6 (42.2 - 45) | 45.1 (43.8 - 46.4) | 40.3 (39.3 - 41.2) | 45.2 (43.9 - 46.5) |
| 2016 | 42.6 (41.3 - 43.9) | 43.8 (42.6 - 45.1) | 39.2 (38.3 - 40.2) | 47.3 (46 - 48.7) |
| 2017 | 43.2 (41.9 - 44.5) | 48.6 (47.2 - 49.9) | 41.4 (40.5 - 42.4) | 48.2 (46.8 - 49.5) |
| 2018 | 44.1 (42.8 - 45.4) | 49.3 (48 - 50.6) | 43.9 (42.9 - 44.8) | 48.2 (46.9 - 49.5) |
| 2019 | 43.1 (41.8 - 44.4) | 50.2 (48.9 - 51.5) | 45.3 (44.3 - 46.3) | 48.5 (47.2 - 49.8) |
| 2020 | 45.8 (44.5 - 47.1) | 52.7 (51.3 - 54) | 46.6 (45.6 - 47.6) | 48.8 (47.5 - 50) |
| 2021 | 49.2 (47.8 - 50.6) | 57.5 (56 - 58.9) | 52.6 (51.6 - 53.7) | 54.3 (52.9 - 55.7) |
| 2022 | 46.6 (45.3 - 48) | 55.2 (53.8 - 56.6) | 51.6 (50.6 - 52.6) | 52.4 (51.1 - 53.7) |
| 2023 | 45.8 (44.4 - 47.1) | 55.9 (54.5 - 57.2) | 52.7 (51.7 - 53.7) | 53.1 (51.8 - 54.4) |
| **Total** | 42.8 (41.4 - 44.2) | 42.8 (41.5 - 44.1) | 38.5 (37.5 - 39.5) | 45.4 (44 - 46.8) |

**Supplemental Table 6.** Region-stratified chronic ischemic heart disease-related age-adjusted mortality rates per 100,000 among older adults with atrial fibrillation in the United States, 1999 to 2023.

| **State** | **Age-Adjusted Rate /100,000 (95% CI)** | **Percentile (%)** |
| --- | --- | --- |
| Georgia | 24.3 (23.7 - 25) | 0 |
| Louisiana | 24.3 (23.5 - 25.2) | 0 |
| Nevada | 26.7 (25.4 - 28) | 4 |
| Hawaii | 26.9 (25.4 - 28.4) | 6 |
| Arkansas | 29.1 (28 - 30.2) | 8 |
| Alabama | 30.1 (29.2 - 31) | 10 |
| Massachusetts | 30.5 (29.7 - 31.2) | 12 |
| Utah | 30.6 (29.1 - 32.1) | 14 |
| Kansas | 30.8 (29.6 - 31.9) | 16 |
| Virginia | 31.3 (30.5 - 32) | 18 |
| Illinois | 31.9 (31.3 - 32.5) | 20 |
| Missouri | 32 (31.2 - 32.8) | 22 |
| Mississippi | 32.3 (31 - 33.5) | 24 |
| Alaska | 32.4 (28.8 - 36) | 26 |
| Wyoming | 33 (30.1 - 35.9) | 28 |
| Montana | 34 (32 - 36) | 30 |
| Florida | 34.1 (33.7 - 34.5) | 32 |
| District of Columbia | 34.9 (32.1 - 37.7) | 34 |
| New Mexico | 34.9 (33.3 - 36.4) | 34 |
| Colorado | 35.7 (34.6 - 36.8) | 38 |
| Arizona | 35.8 (35 - 36.7) | 40 |
| Kentucky | 38 (36.9 - 39.1) | 42 |
| New York | 38.3 (37.8 - 38.8) | 44 |
| South Carolina | 38.5 (37.4 - 39.6) | 46 |
| Wisconsin | 39.2 (38.3 - 40.1) | 48 |
| Texas | 40 (39.5 - 40.5) | 50 |
| South Dakota | 40.2 (37.9 - 42.5) | 52 |
| Michigan | 40.4 (39.7 - 41.2) | 54 |
| Connecticut | 40.5 (39.4 - 41.6) | 56 |
| North Carolina | 41.9 (41.1 - 42.6) | 58 |
| Indiana | 42.2 (41.3 - 43.2) | 60 |
| Nebraska | 42.7 (41 - 44.3) | 62 |
| Idaho | 43 (41.1 - 45) | 64 |
| Maine | 43.5 (41.7 - 45.4) | 66 |
| New Jersey | 43.7 (42.9 - 44.5) | 68 |
| Minnesota | 44.7 (43.7 - 45.8) | 70 |
| Tennessee | 46.5 (45.5 - 47.5) | 72 |
| North Dakota | 46.9 (44.2 - 49.5) | 74 |
| Iowa | 47.2 (45.9 - 48.4) | 76 |
| Oregon | 47.5 (46.3 - 48.8) | 78 |
| California | 48.4 (47.9 - 48.8) | 80 |
| Pennsylvania | 48.5 (47.9 - 49.2) | 82 |
| Maryland | 49.3 (48.2 - 50.4) | 84 |
| New Hampshire | 49.4 (47.3 - 51.6) | 86 |
| Oklahoma | 51.9 (50.6 - 53.3) | 88 |
| Delaware | 52.9 (50.2 - 55.6) | 90 |
| Ohio | 56 (55.2 - 56.7) | 92 |
| Washington | 59.8 (58.7 - 60.9) | 94 |
| West Virginia | 60.3 (58.4 - 62.2) | 96 |
| Vermont | 67.6 (64.1 - 71.1) | 98 |
| Rhode Island | 68.9 (66.3 - 71.4) | 100 |

**Supplemental Table 7.** State-stratified chronic ischemic heart disease-related age-adjusted mortality rates per 100,000 and their respective percentiles among older adults with atrial fibrillation in the United States, 1999 to 2020.
